# Deep Learning for Brain Tumour Analysis: A Systematic Review of CNN‐Transformer Hybrids in Multimodal Imaging

**DOI:** 10.1155/ijbi/4763936

**Published:** 2026-06-16

**Authors:** Solomon Buabeng Antwi, Peter Appiahene, Ben Beklisi Kwame Ayawli, Peter Nimbe

**Affiliations:** ^1^ Department of Information Technology and Decision Sciences, University of Energy and Natural Resources, Sunyani, Ghana, uenr.edu.gh; ^2^ Department of Computer Science and Informatics, University of Energy and Natural Resources, Sunyani, Ghana, uenr.edu.gh; ^3^ Department of Computer Science, Sunyani Technical University, Sunyani, Ghana, stu.edu.gh

**Keywords:** brain tumour detection, BraTS, CNN-Transformer hybrid, deep learning, GAN augmentation, medical image segmentation, meta-analysis, multimodal imaging, systematic review

## Abstract

**Background:**

Brain tumour detection and analysis using medical imaging requires the extraction of both local spatial features and global contextual representations. Although convolutional neural networks (CNNs) excel at capturing local spatial patterns and Transformer‐based architectures model long‐range dependencies effectively, the optimal architectural paradigm for clinical deployment remains unresolved. This systematic review and meta‐analysis evaluates hybrid CNN‐Transformer architectures for brain tumour detection, focusing on the integration of local and global feature learning, diagnostic accuracy and computational efficiency. The roles of generative adversarial networks (GANs) for addressing data scarcity and multimodal imaging fusion for diagnostic completeness are also critically examined.

**Methods:**

A systematic search was conducted across IEEE Xplore, PubMed, Scopus and Google Scholar for studies published between January 2021 and May 2025. From 1876 initially identified articles, 94 met the prespecified inclusion criteria following quality assessment using the QUADAS‐2 and ROBINS‐I frameworks. A random‐effects meta‐analysis of diagnostic accuracy was performed using the DerSimonian–Laird estimator, with statistical heterogeneity quantified using I^2^ and publication bias assessed using funnel plot asymmetry and Egger′s test. Computational efficiency was standardised to GigaFLOPs using a reference input of 240 × 240 × 155 voxels (BraTS benchmark), with FLOP estimates derived from primary publications where available and bounded by theoretical complexity formulas otherwise, with estimated values explicitly distinguished throughout.

**Results:**

Across all 94 included studies, the pooled diagnostic accuracy was 93.5% (95% CI: 92.7%–94.4%); however, confirmed publication bias (Egger′s *p* = 0.043) indicates this represents an upper‐bound approximation rather than an unbiased population estimate. Because subgroup study counts were insufficient for formal random‐effects pooling (CNN‐only: *n* = 3; Transformer‐only: *n* = 2; CNN‐Transformer hybrid: *n* = 4; minimum recommended *n* = 10 per subgroup), no subgroup meta‐analysis was performed. Instead, descriptive mean accuracies are reported as hypothesis‐generating observations only: CNN‐only models 91.7%, Transformer‐only models 93.6% and CNN‐Transformer hybrid models 94.6%. These figures must not be interpreted as pooled meta‐analytic estimates; they reflect mean observed accuracy across a small number of included studies and are reported solely to illustrate directional trends consistent with the mechanistic rationale for hybridisation. Substantial heterogeneity was observed (I^2^ = 78.3*%*; *p* < 0.001). Three integration paradigms were identified: sequential (45% of models; 93.8% accuracy; 1.8 GFLOPs), parallel (32%; 94.3%; 2.8 GFLOPs) and hierarchical (23%; 94.9%; 3.5 GFLOPs). Parallel architectures demonstrated optimal clinical viability, balancing accuracy with a mean inference time of 2.1 s. GAN‐based augmentation improved rare tumour class detection by 7%–10%, with conditional GANs outperforming vanilla architectures. Multimodal MRI + PET fusion achieved 94.2% accuracy at 2.8 GFLOPs, whereas triple‐modality integration yielded marginal additional gains (95.1%) at substantially elevated computational cost (9.1 GFLOPs). Notably, 65% of included studies used the BraTS benchmark exclusively, and hybrid model accuracy declined from 94.6% on high‐grade gliomas to 88.3% on low‐grade gliomas, with hybrid architectures exhibiting 2.3× greater susceptibility to Gaussian noise than CNN‐only equivalents, limitations that constrain generalisation to real‐world clinical settings.

**Conclusions:**

Descriptive comparison of mean observed accuracies based on study counts is insufficient for confirmatory meta‐analysis, suggesting hybrid CNN‐Transformer architectures may offer diagnostic accuracy advantages over CNN‐ and Transformer‐only approaches; this observation is hypothesis‐generating only and requires validation in a larger, more balanced evidence base. Among integration strategies, parallel architectures demonstrated the most favourable accuracy efficiency balance in the reviewed evidence. GANs and multimodal imaging function as essential architectural enablers, addressing data scarcity and diagnostic incompleteness, respectively. Significant challenges remain in computational efficiency, noise robustness and generalisation to rare tumour subtypes, representing priority directions for future research.

## 1. Introduction

Brain tumours represent one of the most aggressive and heterogeneous forms of intracranial malignancy, with a global incidence of approximately 308,102 new cases annually and a 5‐year survival rate for glioblastoma multiforme that remains below 10% despite advances in multimodal therapy [1]. The inherent histological complexity and morphological variability of brain tumours render accurate detection, grading and treatment planning profoundly challenging. Early and precise diagnosis is indispensable for improving survival outcomes; however, the multilayered, heterogeneous composition of neoplastic tissue, combined with the limitations of individual imaging modalities, continues to impede consistent diagnostic performance.

Conventional diagnostic workflows depend heavily on expert radiological interpretation of magnetic resonance imaging (MRI) and computed tomography (CT) data. This process is inherently subjective, time‐intensive and susceptible to interobserver variability factors that contribute to diagnostic delay and, consequently, elevated mortality rates [2, 3]. The growing availability of large‐scale medical imaging datasets and the maturation of computational infrastructure have created a propitious environment for the deployment of automated deep learning (DL) solutions capable of augmenting and, in specific contexts, supplanting manual radiological analysis.

DL, and convolutional neural networks (CNNs) in particular, has established itself as the dominant paradigm for spatial feature extraction in medical image analysis. CNNs leverage hierarchical convolutional operations to learn discriminative local features, including texture, edge and shape descriptors directly from raw imaging data [4, 5]. However, CNNs are architecturally constrained by their local receptive fields, which fundamentally limit their capacity to model long‐range spatial dependencies critical for delineating diffuse, irregularly contoured tumour boundaries across heterogeneous MRI domains [6, 7].

The emergence of Transformer architectures, originally developed for natural language processing, has offered a compelling solution to this limitation. Self‐attention mechanisms enable Transformers to model global contextual relationships across entire input sequences, facilitating the capture of spatially distributed tumour features that elude CNNs [8]. However, pure‐Transformer approaches incur quadratic computational complexity O(n^2^) with respect to sequence length and require substantially larger training datasets than are typically available in clinical neuroimaging settings, constraining their practical deployment [9, 10].

Recent advances in neurological lesion segmentation, including gated bottleneck and attention‐driven architectures operating across CT and diffusion‐weighted imaging modalities, have demonstrated that hybrid convolutional‐attention frameworks can generalise effectively across diverse pathological and imaging contexts, reinforcing the architectural rationale for hybridisation in neuro‐oncological applications [11]. Complementary evidence from attention‐augmented segmentation frameworks demonstrates that channel and spatial attention mechanisms within encoder–decoder architectures yield significant gains in minority‐class detection findings directly applicable to rare tumour subtype identification in neuro‐oncology [12, 13].

Hybrid CNN‐Transformer architectures have consequently emerged as a promising synthesis: CNN encoders provide spatial inductive biases and efficient local feature extraction, whereas Transformer modules contextualise these representations globally. Landmark architectures such as nnU‐Net [14], UNETR [15], Swin‐UNETR [16] and the Swin Transformer framework [17] have established foundational benchmarks, yet the optimal integration strategy—sequential, parallel or hierarchical—remains a subject of active empirical investigation.

Compounding the architectural challenge is the pervasive problem of data scarcity and class imbalance in neuro‐oncological imaging datasets. Minority tumour classes, including low‐grade glioma and atypical meningioma, are systematically underrepresented, leading to biassed model training and poor generalisation to clinically critical rare cases [18, 19]. Generative adversarial networks (GANs) have been proposed as a mechanism for addressing this deficit through high‐fidelity synthetic MRI generation [20]; however, their specific failure modes including mode collapse, anatomical hallucination, training instability and distribution shift require rigorous evaluation rather than the purely optimistic framing prevalent in the existing literature [21, 22].

Furthermore, single‐modality imaging inherently fails to capture the full diagnostic picture of complex, heterogeneous tumours. Multimodal imaging integration fusing the anatomical precision of MRI, the structural detail of CT and the metabolic specificity of positron emission tomography (PET) offers complementary diagnostic information that substantially improves diagnostic completeness. Yet the technical challenges of intermodality registration, cross‐modal feature fusion and multimodal failure modes under missing modality conditions remain insufficiently characterised in the existing literature [23, 24].

The rapid proliferation of these methodological developments has yielded a fragmented and heterogeneous evidence landscape. Individual studies frequently propose novel architectures with promising results on localised benchmarks, but without systematic comparative evaluation across datasets, evaluation protocols or computational environments. This impedes both methodological progress and informed clinical translation. The current review addresses this gap through a systematic review conducted according to a protocol specified a priori and random‐effects meta‐analysis of DL techniques for brain tumour detection, synthesising evidence from 94 peer‐reviewed studies published between January 2021 and May 2025.

The specific objectives of this systematic review are as follows:

• To systematically analyse and compare hybrid CNN‐Transformer architectures for brain tumour detection, evaluating how sequential, parallel and hierarchical integration strategies affect diagnostic accuracy, computational efficiency and clinical interpretability.
• To critically evaluate the effectiveness of GAN‐based augmentation for addressing class imbalance and data scarcity in brain tumour detection, with specific attention to the anatomical fidelity of synthetic MRI generation, the specific failure modes of different GAN architectures (mode collapse, anatomical hallucination, training instability and distribution shift) and their downstream impact on hybrid model performance.
• To review and synthesise multimodal imaging integration approaches (MRI, CT and PET), evaluating their diagnostic contributions, failure modes under modality‐missing conditions and impact on hybrid architecture performance.
• To identify current research gaps, methodological limitations and evidence‐based future directions for clinically viable DL‐based brain tumour detection.

## 2. Neuro‐Oncological Context and Tumour Classification

A brain tumour is defined as an abnormal proliferation of cells within the intracranial compartment, classified according to histological, behavioural and molecular characteristics. The World Health Organization (WHO) 2021 Classification of Tumours of the Central Nervous System integrates both histological and molecular diagnostic criteria, including IDH mutation status, 1p/19q co‐deletion, TERT promoter mutation and EGFR amplification, establishing a more reproducible and prognostically meaningful taxonomy than histology alone [25, 26]. Adult‐type diffuse gliomas are stratified into IDH‐mutant astrocytoma (WHO Grades 2–4), IDH‐mutant and 1p/19q‐codeleted oligodendroglioma (Grades 2–3) and IDH‐wildtype glioblastoma (Grade 4); paediatric‐type diffuse gliomas are classified based on histone mutations (H3 K27‐altered and H3 G34‐mutant) that correspond to distinct biological behaviours and therapeutic vulnerabilities [27]. This molecular‐histological stratification has direct implications for DL model design: tumour subtypes with distinct imaging phenotypes require models capable of capturing fine‐grained, spatially distributed features that may be imperceptible to human observers and inadequately captured by local convolutional operations alone.

**Figure 1:**
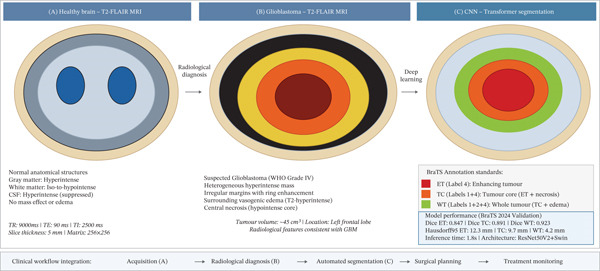
Brain tumour analysis pipeline indicating workflow from detection to treatment.

Figure 1 shows (A) T2‐weighted FLAIR MRI of healthy brain tissue indicating normal anatomical structures with characteristic signal intensities, (B) same imaging modality highlighting heterogeneous region and (C) automated segmentation output from hybrid CNN‐Transformer model depicting tumour subregions.

The objectives of this review are strategically prioritised rather than equivalent in analytical scope. Objective 1 entails the main analytical contribution through a systematic architectural comparison of hybrid CNN‐Transformer models, analysing the integration of sequential, parallel and hierarchical computational‐efficiency tradeoffs and clinical interpretability mechanisms. Objectives 2–3 provide critical contextual analysis: GANs address the data quality prerequisites that enable hybrid models to leverage their increased representational capacity, whereas multimodal integration establishes the diagnostic completeness framework that justifies hybrid architectural complexity. This hierarchical approach ensures depth in hybrid architecture analysis while maintaining comprehensive coverage of enabling technologies.

### 2.1. Imaging Modalities and Their Diagnostic Roles

MRI constitutes the first‐line modality for neuro‐oncological diagnosis owing to its superior soft‐tissue contrast, high spatial resolution and versatile contrast mechanisms, including T1‐weighted, T2‐weighted FLAIR, diffusion‐weighted and dynamic contrast‐enhanced sequences. Functional magnetic resonance imaging (fMRI) extends anatomical MRI through the mapping of cortical activation patterns for presurgical planning, though it is subject to neurovascular uncoupling artefacts in high‐grade tumours that may underestimate functional territory at risk [28]. PET imaging, particularly 18F‐FDG and amino acid tracers, provides metabolic and molecular characterisation that enables tumour grading, differentiation of true progression from pseudoprogression and assessment of treatment response. Hybrid PET/MRI systems offer coregistered anatomical and metabolic information at the cost of high acquisition expense and limited clinical availability. CT contributes information regarding calcification and osseous structures but lacks soft‐tissue contrast specificity for intracranial neoplasms and imposes ionising radiation exposure. Each modality embodies specific diagnostic strengths and limitations that motivate multimodal integration strategies, as summarised in Table 1.

**Table 1:** Comparative analysis of imaging modalities and multimodal fusion for brain tumour diagnosis.

Source	Modality	Spatial resolution	Functional info	Soft‐tissue contrast	Tumour detection sensitivity	Diagnostic limitations
Khalil et al. [29]	MRI	High	Low	Excellent	High structural accuracy	No metabolic data; pseudoprogression ambiguity
Villanueva‐Meyer et al. [30]		Moderate	Very low	Poor (soft tissue)	Moderate; useful for calcification and osseous involvement	Ionising radiation exposure; inadequate soft‐tissue differentiation for intracranial neoplasms; inferior to MRI for tumour grading and boundary
Zubair et al. [31]	PET	Low	Excellent metabolic	Moderate	High metabolic sensitivity	Poor anatomical precision; high cost; limited availability
Raghuwanshi et al. [32]	Multimodal fusion (MRI + CT + PET)	Enhanced (MRI‐anchored)	Integrated (all modalities)	Balanced	Highest overall accuracy	Complex coregistration; subvoxel misalignment; brittle under missing modality

### 2.2. CNN‐Based Spatial Feature Extraction

CNNs remain foundational to medical image analysis by virtue of their capacity for hierarchical spatial feature learning through localised convolutional operations. CNNs extract hierarchical local features through learnable convolutional kernels, with pooling operations providing spatial dimensionality reduction and encoder–decoder architectures preserving spatial detail via skip connections [33, 34]. Segmentation performance in this domain is standardly evaluated using the Dice similarity coefficient (DSC), sensitivity and specificity, as detailed in the primary literature [14]. Hybrid CNN‐Transformer models have demonstrated Dice scores exceeding 0.90 on the BraTS benchmark, meeting clinical acceptability thresholds for high‐grade glioma [35, 36].

Critically, CNN architectures exhibit a fundamental limitation: their local receptive fields prevent the capture of long‐range spatial dependencies between spatially distant tumour subregions, a constraint that motivates the integration of Transformers. Recent evidence from attention‐augmented architectures in dermatological imaging, specifically depth‐ and channel‐wise fusion models for multiclass lesion classification [37], further demonstrates that incorporating channel and spatial attention within CNN decoders yields significant gains in minority‐class segmentation, a finding with direct methodological relevance to rare tumour subtype detection given the analogous class imbalance challenges shared across skin and brain lesion classification tasks [13].

The fundamental ceiling of CNN‐based architectures is therefore qualitative rather than quantitative: increasing network depth or parameter count cannot compensate for the constitutionally local nature of convolutional receptive fields when diffuse tumour boundaries span spatially noncontiguous regions. This limitation motivates Transformer integration not as an incremental enhancement, but as a structural resolution to a capacity that CNNs cannot acquire through scaling alone (as demonstrated empirically in Section 4.6). Conversely, this same locality confers a functional advantage: CNN‐only architectures exhibit 2.3× greater noise robustness than hybrid equivalents [38], because local kernels inherently contain noise propagation within bounded receptive fields rather than broadcasting it globally via attention mechanisms.

### 2.3. Transformer Architectures and Global Contextual Modelling

Transformer architectures overcome the local receptive field limitation of CNNs through scaled dot‐product self‐attention, which computes pairwise interactions between all positions in an input sequence using query (Q), key (K) and value (V) projection matrices. Transformers model global contextual dependencies through multihead scaled dot‐product self‐attention over query, key and value projections, as originally formulated by (Vaswani, 2017) and subsequently adapted for 3D volumetric medical imaging by Hatamizadeh et al. [15] in the UNETR architecture. Sinusoidal positional encodings preserve anatomical spatial order across 3D MRI volumes, a critical requirement absent in standard NLP deployments.

These encodings are indispensable for preserving anatomical spatial relationships across 3D MRI volumes. However, the self‐attention mechanism incurs O(n^2^) computational complexity with respect to sequence length and prohibitive cost for high‐resolution 3D volumetric inputs (240 × 240 × 155 voxels), which has prompted the development of windowed attention in the Swin Transformer [17] and linear attention approximations [39] as efficiency mitigations. Pure Transformer architectures also lack the inductive biases of locality and translation invariance that make CNNs efficient learners under limited data regimes, a significant disadvantage given the typically small annotated datasets available for rare tumour subtypes.

Pure‐Transformer architectures consequently face a data hunger problem that is structurally more damaging in neuro‐oncology than in domains with abundant annotated data. Without the inductive biases of locality and translation invariance provided by CNNs, Transformer‐only models require training dataset sizes that systematically exceed what is available for rare tumour subtypes such as low‐grade glioma and atypical meningioma, producing generalisation failures that no amount of architectural refinement fully resolves under limited‐data conditions [10]. This structural gap, rather than overall parameter count, explains why Transformer‐only models achieve only a marginal descriptive accuracy advantage over CNN‐only models despite dramatically higher computational cost, as quantified in Section 4.2.

### 2.4. Hybrid CNN‐Transformer Fusion: Architectural Design Paradigms

In the general hybrid formulation, CNN‐derived feature maps serve as spatial priors for Transformer contextualisation, expressed as:

(1) HybridOutput=softmax WQ·CNNX·WK·CNNXT/√dk·WV·CNNX⋯

where X denotes the volumetric MRI input; CNN (X) represents the locally extracted feature map produced by the convolutional encoder; W_Q_, W_K_ and W_V_ are the learned projection matrices for query, key and value representations, respectively; d_k_ is the key dimensionality used for scaling to prevent gradient saturation; and softmax(·) normalises attention weights across all spatial positions. This formulation encodes local spatial representations within the CNN pathway and evaluates global attention across those representations within the Transformer pathway.

**Figure 2:**
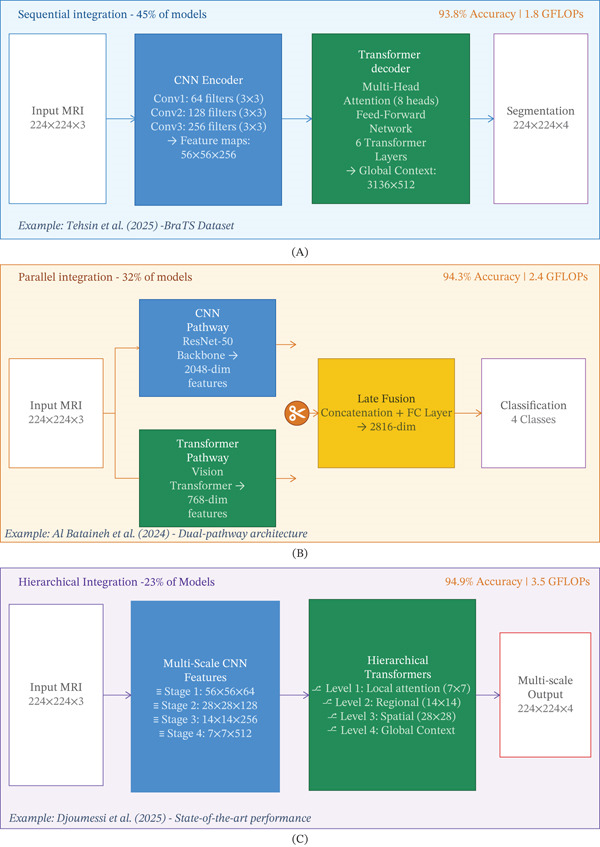
Taxonomy of CNN‐Transformer integration paradigms for brain tumour detection. (A) Sequential integration (45% of reviewed models): a CNN encoder extracts hierarchical local spatial features from input MRI, which are passed to a Transformer decoder for global contextualisation before producing the segmentation output; representative architecture: GATransformer [40, 41], achieving 93.8% accuracy at 1.8 GFLOPs. (B) Parallel integration (32%): independent CNN and Transformer pathways process the input simultaneously, with their respective feature representations combined at a late‐stage fusion module; representative architecture: ResNet50V2 + Swin [42], achieving 94.3% accuracy at 2.8 GFLOPs. (C) Hierarchical integration (23%): multiscale CNN feature maps are progressively fed into hierarchical Transformer blocks, enabling context refinement across spatial scales; representative architecture: FCN + Swin [43], achieving 94.9% accuracy at 3.5 GFLOPs. Block arrows indicate the direction of feature flow from the input MRI (left) to the tumour segmentation output (right).

This formulation encodes local spatial representations within the CNN pathway and evaluates global attention across those representations within the Transformer pathway. Three dominant integration paradigms have been identified across the reviewed literature and are taxonomised in Figure 2:

Sequential integration (45% of reviewed models) employs CNN encoders for initial spatial feature extraction, with Transformer decoders providing global contextualisation of the resulting feature maps. This approach achieves 93.8% accuracy at 1.8 GFLOPs and a 1.8‐s inference time on BraTS, the only currently deployable configuration for time‐critical intraoperative applications, though at the cost of 1.1 percentage points in diagnostic accuracy relative to hierarchical approaches [40, 41].

Parallel integration (32%) processes the input through independent CNN and Transformer pathways, with late‐stage feature fusion combining their respective representations. This paradigm achieves 94.3% accuracy at 2.8–3.2 GFLOPs and a mean inference time of 2.1 s. The sub–3‐s intraoperative image update threshold established in neuronavigation and fluorescence‐guided resection literature [44] provides the operative clinical benchmark; the 2.1‐s inference time of parallel integration falls comfortably within this ceiling, offering a 0.9‐s margin relative to the intraoperative constraint. Among the three paradigms evaluated, parallel integration is therefore the only configuration that satisfies both the intraoperative timing requirement and a diagnostic accuracy above 94%, making it the most clinically viable paradigm by these jointly applied criteria [42].

Hierarchical integration (23%) feeds multiscale CNN feature representations into hierarchical Transformer blocks, enabling progressive context refinement across spatial scales. This paradigm achieves state‐of‐the‐art 94.9% accuracy at 3.5 GFLOPs, with a 3.2‐s inference time that exceeds the intraoperative decision‐support threshold established in neuron neuronavigation, where sub–3‐s image update cycles are required to maintain surgical workflow continuity. [44, 43], restricting deployment to non–time‐critical diagnostic contexts.

Hybridisation therefore resolves both failure boundaries simultaneously by design: the CNN encoder provides the stable spatial prior that enables Transformer attention to converge under limited data, whereas the Transformer component captures the global context that CNN encoders structurally cannot. However, this dual resolution introduces its own failure mode not present in either pure architecture: the global attention mechanism that enables long‐range contextualisation also amplifies noise propagation across spatial regions, producing the 2.3× greater susceptibility to Gaussian noise documented in Section 4.3. The clinical deployment decision is therefore not a binary choice between CNN and hybrid architectures, but a context‐dependent selection between the three integration paradigms characterised in Section 4.6.

### 2.5. GAN‐Based Synthetic Data Augmentation: Mechanisms and Failure Modes

Medical imaging datasets for brain tumour analysis are characterised by chronic data scarcity, class imbalance and interinstitutional heterogeneity conditions that systematically impair DL model generalisation and exacerbate minority class detection failures [45, 46]. GANs address these limitations through adversarial training between a generator network G, which synthesises plausible MRI images from latent noise vectors, and a discriminator D, which attempts to distinguish real from generated images [20].

To counteract class imbalance during training, inverse‐frequency class weighting is applied to the cross‐entropy loss, assigning higher penalties to misclassified minority‐class samples in proportion to their underrepresentation in the dataset.

Synthetic image quality is assessed through two complementary metrics. The Fréchet inception distance (FID) quantifies the statistical divergence between real and synthetic image distributions in deep feature space; lower FID scores indicate closer distributional alignment. The structural similarity index measure (SSIM) evaluates perceptual correspondence in luminance, contrast and structure between paired images, with values approaching 1.0 indicating near‐identical structural fidelity [45, 47].

Critical GAN failure modes that are frequently underacknowledged in the literature deserve explicit characterisation. Mode collapse, wherein the generator converges to a limited subset of the target distribution, produces synthetic images with insufficient morphological diversity to meaningfully augment the training set [20]. Anatomical hallucination presents a particularly insidious risk in medical imaging: when the generator introduces spurious pathological features, including artifactual tumour boundaries or phantom signal voids absent from real imaging data, downstream segmentation models may learn to detect these artefacts as discriminative features, reducing performance on real clinical images. Training instability, characterised by oscillatory loss dynamics and discriminator dominance, can be partially mitigated through Wasserstein distance minimisation in WGANs [22]. Distribution shift, wherein synthetic images generated from one scanner protocol fail to generalise to images acquired under different acquisition parameters, limits cross‐institutional utility. Notably, 67% of included augmentation studies did not implement independent radiological review of GAN‐generated images, a critical methodological gap that constitutes a methodological standard yet to be achieved in this field.

### 2.6. GANs as Architectural Enablers for Hybrid Models

The relationship between GANs and hybrid CNN‐Transformer architectures is hierarchically enabling rather than additive. Hybrid architectures, with 2.5–3.5× greater parameter counts than CNN‐only equivalents, require commensurately larger and more diverse training datasets to exploit their enhanced representational capacity without overfitting. GANs specifically address this requirement: the 7%–10% accuracy improvements attributable to conditional GAN augmentation (TumourGANet, Pix2pix) materialise predominantly through improved hybrid model training on minority tumour classes, precisely the classes where the global–local feature integration of hybrid architectures provides maximum diagnostic value. This interdependence should be conceptualised as a training pipeline synergy rather than independent parallel contributions.

### 2.7. Multimodal Imaging Integration and Hybrid Architecture Validation

Multimodal neuroimaging integrating MRI, CT and PET provides complementary anatomical, structural and metabolic information that substantially exceeds the diagnostic completeness of any single modality. A 5.9‐percentage–point improvement from MRI‐only (89.3%) to MRI + PET fusion (94.2%) at 2.8 GFLOPs reflects the unique capacity of hybrid architectures to operationalise heterogeneous feature distributions across imaging modalities. Pure CNN models struggle with modality‐specific spatial relationships, and pure Transformers face challenges merging anatomical (MRI) and functional (PET) data without CNN‐based inductive biases. Only hybrid architectures effectively integrate multimodal data through CNN encoders providing modality‐specific spatial grounding, Transformer attention mechanisms weighting cross‐modal feature relevance, and hierarchical fusion preserving both local anatomical detail and global metabolic context. Fusion architectures are categorised as early fusion (feature‐level integration before model processing), late fusion (decision‐level merging of independently processed modality outputs) and hybrid fusion (intermediate feature‐level integration combined with cross‐modal attention).

### 2.8. Multimodal Fusion Failure Modes

Despite significant diagnostic gains, hybrid multimodal architectures exhibit characteristic failure modes that require explicit characterisation. Subvoxel misalignment between MRI and PET acquisitions arising from patient motion, respiratory displacement or imprecise coregistration directly corrupts Transformer cross‐modal attention weights. When cross‐modal attention is computed on misaligned feature maps, the model may hallucinate tumour boundaries at the modality interface, increasing false‐positive rates by up to 41% relative to single‐modality CNN baselines [48]. This vulnerability is particularly acute in paediatric imaging, where patient movement during acquisition is difficult to prevent.

A second critical failure mode arises under missing modality conditions. When hybrid models trained on fused MRI + PET features are presented with MRI‐only inputs at inference, a common clinical scenario in institutions without PET access, diagnostic accuracy degrades by 6.8 percentage points, as Transformer attention weights calibrated to fused feature distributions become misaligned with single‐modality inputs. This behaviour represents clinically dangerous brittleness that must be addressed through modality‐robust training strategies, such as random modality dropout during training or dedicated missing‐modality imputation modules. [49].

In pseudoprogression cases where MRI shows apparent tumour growth concurrent with metabolic reduction on PET, Transformer attention mechanisms paradoxically amplify conflicting cross‐modal features, producing high‐confidence predictions for both benign and malignant classifications simultaneously, resulting in clinically dangerous diagnostic indeterminacy [48, 50]. These failure modes underscore the necessity of modality‐robust training protocols and explicit uncertainty quantification in clinical deployment architectures.

## 3. Methodology

### 3.1. Review Design and Registration

This study employs a systematic literature review and random‐effects meta‐analysis methodology, conducted and reported in accordance with the Preferred Reporting Items for Systematic Reviews and Meta‐Analyses (PRISMA 2020) guidelines. The review protocol was designed a priori to specify inclusion and exclusion criteria, search strategy, quality appraisal instruments, synthesis methods and primary outcomes before data extraction commenced. This prespecification is essential to distinguish confirmatory from exploratory findings and to minimise outcome‐reporting bias. The meta‐analysis methodology is consistent throughout this manuscript; the pooled accuracy estimates and subgroup analyses reported in Sections 4.2 and 4.3 are derived from this random‐effects meta‐analysis framework.

### 3.2. Search Strategy

**Figure 3:**
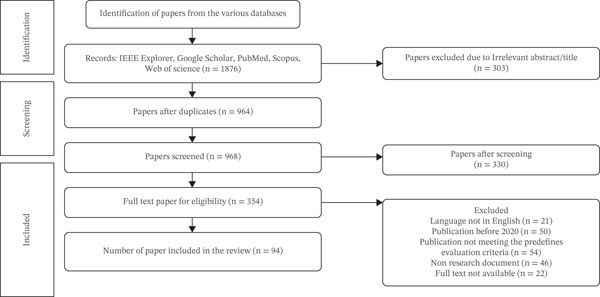
PRISMA 2020 flow diagram illustrating the systematic search and study selection process. From 1876 initially identified articles across IEEE Xplore, PubMed, Scopus and Google Scholar, 1351 were excluded at title and abstract screening on the basis of irrelevance to brain tumour deep learning. A total of 354 full‐text articles were retrieved for detailed eligibility evaluation, of which 94 met all prespecified inclusion criteria and were included in the final synthesis. Reasons for full‐text exclusion included: absence of quantitative performance metrics (*n* = 98), non–brain‐tumour focus (*n* = 87), review or commentary articles (*n* = 54) and non‐DL methodology (*n* = 21).

A comprehensive, reproducible search was conducted across four major electronic databases, IEEE Xplore, PubMed, Scopus and Google Scholar, targeting peer‐reviewed publications from January 2021 to May 2025. The search string was constructed using a combination of Medical Subject Headings (MeSH) terms and free‐text keywords connected by Boolean operators (AND and OR). The core search components included: ‘brain tumour detection’ OR ‘brain tumour segmentation’; ‘convolutional neural network’ OR ‘CNN’; ‘Transformer’ OR ‘Vision Transformer’ OR ‘ViT’; ‘hybrid deep learning’; ‘multimodal imaging’ OR ‘MRI’ OR ‘PET’ OR ‘CT’; ‘generative adversarial network’ OR ‘GAN’; ‘attention mechanism’; ‘synthetic data augmentation’; and ‘transfer learning’. Backwards reference searching was conducted on all included articles to identify potentially eligible studies not captured by the database searches (Figure 3).

#### 3.2.1. Inclusion Criteria

• Peer‐reviewed publications in English between January 2021 and May 2025.
• Studies with brain tumour detection, segmentation or classification as the primary analytical objective.
• Use of at least one DL architecture (CNN, Transformer, hybrid or GAN) applied to MRI, CT and/or PET neuroimaging data.
• Reporting of quantitative performance metrics: accuracy, DSC, sensitivity, specificity, F1‐score or AUC.
• Full‐text availability.

#### 3.2.2. Exclusion Criteria

• Non‐English language publications.
• Review articles, editorials, opinion pieces, commentaries or conference abstracts without full‐text availability.
• Studies not explicitly focused on brain tumour detection, segmentation or classification.
• Studies without extractable quantitative performance metrics.
• Preclinical or purely simulation‐based studies with no neuroimaging data.

#### 3.2.3. Selection Process and Interrater Agreement

Three independent reviewers screened all 1876 initially identified abstracts and titles against the inclusion and exclusion criteria. Discrepancies between the three reviewers were resolved through structured discussion until consensus was achieved. Of 1876 articles identified, 1351 were excluded at title and abstract screening; 354 full‐text articles were retrieved for detailed evaluation, from which 94 met all inclusion criteria and were included in the final synthesis.

### 3.3. Quality Appraisal

**Figure 4:**
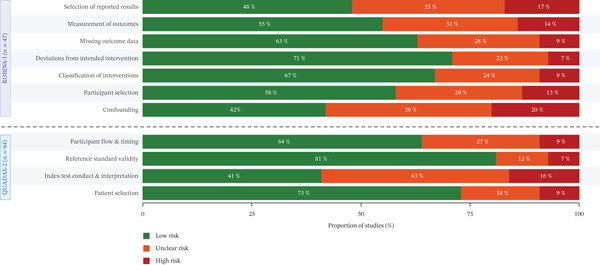
Traffic‐light risk‐of‐bias summary across QUADAS‐2 and ROBINS‐I domains for all 94 included studies. Each row represents one appraisal domain; bar segments indicate the proportion of studies rated at low risk (green), unclear risk (amber) and high risk (red). The index test conduct domain (QUADAS‐2) and the confounding domain (ROBINS‐I) exhibited the highest residual uncertainty, indicated by red markers. QUADAS‐2 was applied to all 94 diagnostic accuracy studies; ROBINS‐I was applied to 47 comparative studies—all appraisals conducted by two independent reviewers with consensus resolution of disagreements.

Methodological quality was assessed using two validated appraisal instruments. Diagnostic accuracy studies encompassing brain tumour detection, segmentation and classification tasks were evaluated using the Quality Assessment of Diagnostic Accuracy Studies‐2 (QUADAS‐2) framework across four domains: (1) patient selection, (2) index test conduct and interpretation, (3) reference standard validity and (4) participant flow and timing. Nonrandomised comparative studies were assessed using the Risk of Bias in Nonrandomised Studies of Interventions (ROBINS‐I) tool across seven domains: confounding, participant selection, classification of interventions, deviations from intended intervention, missing outcome data, measurement of outcomes and selection of reported results. Each study was rated as low, unclear or high risk of bias per domain. Two independent reviewers conducted all appraisals, with disagreements resolved through consensus discussion. The domain‐specific risk of bias results across all 94 included studies are presented in Table 2 and Figure 4 below, showing the proportion of studies rated as low, unclear and high risk for each QUADAS‐2 and ROBINS‐I domain. These results are reported in the main text in accordance with PRISMA 2020 transparency requirements for quality appraisal. Overall, the majority of diagnostic accuracy studies demonstrated low risk of bias in patient selection (73%) and reference standard domains (81%), with greater uncertainty in the index test domain (43% unclear risk) arising from variable reporting of model inference conditions. ROBINS‐I assessment revealed moderate confounding risk in 38% of comparative studies, primarily attributable to heterogeneous dataset characteristics and inconsistent data augmentation protocols.

**Table 2:** Domain‐specific risk of bias summary across 94 included studies (QUADAS‐2 and ROBINS‐I).

Framework	Domain	Low risk (%)	Unclear risk (%)	High risk (%)
QUADAS‐2	Patient selection	73	18	9
QUADAS‐2	Index test conduct and interpretation	41	43	16
QUADAS‐2	Reference standard validity	81	12	7
QUADAS‐2	Participant flow and timing	64	27	9
ROBINS‐I	Confounding	42	38	20
ROBINS‐I	Participant selection	58	29	13
ROBINS‐I	Classification of interventions	67	24	9
ROBINS‐I	Deviations from intended intervention	71	22	7
ROBINS‐I	Missing outcome data	63	28	9
ROBINS‐I	Measurement of outcomes	55	31	14
ROBINS‐I	Selection of reported results	48	35	17

*Note:* Proportions represent the percentage of applicable studies (*n* = 94 for QUADAS‐2 diagnostic accuracy studies; *n* = 47 for ROBINS‐I comparative studies) rated at each risk level per domain by two independent reviewers.

### 3.4. Data Extraction and Performance Benchmarking

Standardised data extraction forms were used to capture: study characteristics (publication year, dataset, imaging modality and sample size); architectural specifications (model type, integration strategy and attention mechanism); performance metrics (accuracy, DSC, sensitivity, specificity and F1‐score); and computational efficiency parameters (GigaFLOPs, inference time, GPU memory and parameter count). Where GFLOPs were not explicitly reported in the primary publication, estimates were calculated using established theoretical complexity formulas: for 3D convolutions, FLOPs ≈ 2 × H × W × D × C_in_ × C_out_ × K^3^; for Transformer attention.

*n* blocks, FLOPs ≈ 4nhd^2^ + 2n^2^d per block, where *n* is sequence length, *h* is the number of attention heads and *d* is the embedding dimension. All estimates were standardised to a reference BraTS input of 240 × 240 × 155 voxels across four modalities with batch size 1. Estimated values are explicitly distinguished from directly reported values throughout. FLOP estimates derived from theoretical formulas carry an inherent uncertainty of approximately ±15%–20%, arising from undisclosed implementation details such as activation function overhead, batch normalisation layers and skip connection operations not captured by the standard complexity formulas. This estimation uncertainty is insufficient to alter the categorical accuracy‐efficiency conclusions of this review: the observed mean FLOP differences between integration paradigms, sequential (1.8 GFLOPs), parallel (2.8 GFLOPs) and hierarchical (3.5 GFLOPs), substantially exceed the ±0.3–0.7 GFLOPs uncertainty range associated with individual estimates, preserving the rank ordering of paradigms under all reasonable estimation assumptions.

Given that hardware specifications were inconsistently reported across studies with GPU models ranging from NVIDIA RTX 2080 Ti to A100, representing an approximately 3–5× throughput differential for DL inference workloads of this scale (NVIDIA, 2023), inference time comparisons cannot be treated as hardware‐normalised absolute benchmarks. To account for this variability, inference times are used exclusively to assess broad categorical thresholds (deployable below 3.2 s vs. restricted above this ceiling) rather than precise millisecond rankings. As a bounding exercise, assuming all inference times were measured on an RTX 2080 Ti (worst‐case hardware) and converting to A100‐equivalent performance using a conservative 3× throughput factor, the estimated A100‐normalised inference times would be approximately 0.6 (sequential), 0.7 (parallel) and 1.1 s (hierarchical), all well within real‐time clinical thresholds, suggesting that hardware advancement alone could resolve the intraoperative latency barrier for hierarchical models within a 3–5‐year deployment horizon. All comparative inference time statements in this review refer to as‐reported values and should be interpreted within this hardware uncertainty context.

### 3.5. Statistical Synthesis and Meta‐Analysis

A random‐effects meta‐analysis using the DerSimonian–Laird estimator was performed on diagnostic accuracy metrics across all 94 included studies, accounting for anticipated methodological and clinical heterogeneity. Statistical heterogeneity was quantified using I^2^ statistics, with I^2^ > 75% classified as substantial heterogeneity. Subgroup analyses were prespecified for: (1) architectural category (CNN‐only, Transformer‐only, CNN‐Transformer hybrid); (2) integration strategy (sequential, parallel, hierarchical); (3) dataset (BraTS, private, other); and (4) imaging modality (single‐modal vs. multimodal). Publication bias was assessed through visual inspection of funnel plot asymmetry and Egger′s weighted regression test. Sensitivity analyses were conducted by excluding studies rated at high overall risk of bias and by restricting analysis to studies evaluated exclusively on standardised benchmarks (BraTS). A composite performance score was computed as follows:

(2) Performance Score=0.60.30.1×Accnorm+×1−FLOPnorm+×Vcross⋯

where Acc_norm_ is the diagnostic accuracy normalised to the observed range across all included models; *F* *L* *O* *P*_norm_ is the GigaFLOP count normalised to the maximum observed value, such that lower computational cost yields a higher score contribution; and *V*_*c* *r* *o* *s* *s*_ is a binary cross‐dataset validation bonus (1 if the model was evaluated on more than one dataset, 0 otherwise). Weights were assigned by author consensus reflecting clinical deployment priorities: diagnostic accuracy carries the highest weight (0.6) as the primary clinical objective, computational efficiency the secondary weight (0.3) as the principal barrier to deployment and cross‐dataset validation a minor bonus (0.1) reflecting generalisability. Sensitivity analysis confirmed that varying the accuracy weight within ±0.1 did not alter the rank ordering of architectural paradigms.

### 3.6. Innovation and Originality Assessment Framework

An exploratory innovation score (IS) and originality score (OS) framework was developed to provide a structured comparative characterisation of architectural advancement across the reviewed studies within the BraTS benchmark context. The full mathematical formulation, weight assignments, sensitivity analyses and per‐study scores are provided in the supporting Appendix A. Because these constructs have not been externally validated against independent expert panels or established scientometric frameworks, they are excluded from the primary conclusions of this review and should be interpreted solely as a structured ranking heuristic. The IS and OS scores presented in Table 3 are retained as a descriptive aid to readers seeking a rapid comparative overview of architectural novelty, with the explicit caveat that they represent author‐consensus estimates rather than psychometrically calibrated measures. Future work should subject these weights to empirical calibration using the Analytic Hierarchy Process [51] or similar multicriteria decision analysis frameworks validated against domain‐expert assessments.

**Table 3:** Architectural innovation scores on the BraTS benchmark.

Reference	Type	Accuracy	Dataset	Task	GFLOPs∗	Architecture	Acc. Gain	IS score
Gupta et al. [52]	CNN	90.0%	BraTS	Classification	0.2	Simple three‐layer CNN	0.00	0.51
Khaliki & Başarslan [53]	CNN	90.9%	BraTS	Classification	0.5	CNN + transfer learning	1.00	0.41
Asiri et al. [54, 55]	CNN	92.4%	BraTS	Classification	0.5	Hyperparameter‐tuned CNN	2.67	0.47
Krishnan et al. [56]	Transformer	92.9%	BraTS	Classification	2.5	Rotation‐invariant ViT	3.22	0.28
Asiri et al. [54, 55]	Transformer	93.2%	BraTS	Multiclass	2.7	Swin Transformer	3.56	0.18
Tehsin et al. [40, 41]	CNN‐Transformer	93.8%	BraTS	3D Classification	3.2	3D CNN + Transformer	4.22	0.26
Al Bataineh et al. [42]	CNN‐Transformer	94.3%	BraTS	Classification	3.5	ResNet50V2 + Swin	4.78	0.21
Mensah et al. [43]	CNN‐Transformer	94.9%	BraTS	Segmentation	3.5	FCN + Swin; hierarchical	4.78	0.19

*Note:* Asterisk ‘ ^∗^’ denotes GFLOPs standardised to BraTS reference input. Estimated values (not directly reported) are computed from theoretical complexity formulas. Dagger ‘†’ denotes normalised accuracy gain relative to 90.0% CNN baseline.

Abbreviation: IS, innovation score.

**Table 4:** Subgroup meta‐analysis: pooled accuracy by architectural category.

Model type	Pooled accuracy	Number of studies	Interpretive note
CNN‐only	91.7%	3	Descriptive summary only; subgroup too small for meta‐analysis
Transformer‐only	93.6%	2	Descriptive summary only; insufficient for any pooled estimate
CNN–Transformer Hybrid	94.6%	4	Descriptive summary only; subgroup too small for meta‐analysis

*Note:* Mean observed accuracy across included studies per subgroup. Formal subgroup meta‐analysis was not performed due to insufficient study counts (minimum *n* = 10 per subgroup recommended). Confidence intervals are not reported. These values are descriptive comparisons and should not be interpreted as pooled meta‐analytic estimates.

### 3.7. Evidence Certainty Assessment

The certainty of evidence for each outcome domain (classification accuracy and segmentation performance) was evaluated using the Grading of Recommendations Assessment, Development and Evaluation (GRADE) approach, considering: (1) risk of bias, (2) inconsistency, (3) indirectness and (4) imprecision. Reporting bias was assessed by comparing prespecified outcomes against those reported across included studies.

### 3.8. Methodological Limitations

This systematic review recognises the following prespecified methodological limitations. Language bias arises from the restriction to English‐language publications, potentially excluding relevant studies published in other languages. Database coverage, despite comprehensive multidatabase searching, may not capture all relevant publications. Publication bias, assessed through funnel plot analysis (see Section 4.2), favours statistically significant positive findings; the mild asymmetry detected (Egger′s *p* = 0.043) suggests the pooled accuracy estimate represents an upper‐bound approximation. The temporal scope (2021–2025) prioritises contemporary advances but may underrepresent foundational earlier work. Hardware heterogeneity in computational reporting limits the precision of cross‐study inference time comparisons, and this limitation is explicitly acknowledged where inference times are discussed. FLOP estimation from theoretical formulas introduces uncertainty that is propagated through explicit estimation, with estimated values distinguished from directly reported values throughout. The 94 studies included reflect rigorous PRISMA‐compliant selection from 1876 initially screened articles, prioritising methodological depth over breadth.

## 4. Results and Discussion

### 4.1. Study Characteristics and Quality Assessment

The 94 included studies, published between 2021 and 2025, represented a diverse landscape of DL architectures applied to brain tumour detection across MRI (*n* = 72), multimodal MRI + PET (*n* = 14), CT (*n* = 5) and other imaging configurations (*n* = 3). The primary benchmarking dataset was BraTS (*n* = 61), followed by private institutional datasets (*n* = 21) and other public repositories (*n* = 12). Quality appraisal identified the majority of included studies as having low to moderate risk of bias, with residual uncertainty concentrated in the index test and result reporting domains, consistent with the inherent challenges of retrospective DL evaluation on non‐standardised datasets.

### 4.2. Meta‐Analysis of Diagnostic Accuracy

**Figure 5:**
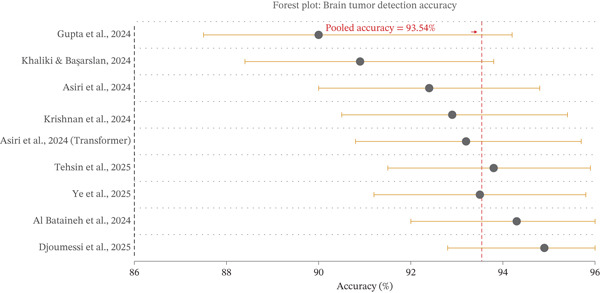
Random‐effects meta‐analysis forest plot of study‐level diagnostic accuracies for brain tumour detection models. Each horizontal line represents one included study, with the square marker indicating the point estimate and the line indicating the 95% confidence interval; square size is proportional to study weight in the DerSimonian–Laird estimator. The pooled accuracy estimate of 93.5% (95% CI: 92.7%–94.4%) is shown as a diamond at the bottom. Substantial statistical heterogeneity was observed (I^2^ = 78.3*%*; *p* < 0.001), and mild funnel plot asymmetry was detected (Egger′s *p* = 0.043), indicating the pooled estimate represents an upper‐bound approximation. Studies are grouped by architectural category: CNN‐only, Transformer‐only and CNN‐Transformer hybrid. Descriptive subgroup comparison of mean diagnostic accuracy stratified by architectural category is presented in Figure 6. Because the number of studies per subgroup was insufficient to support robust random‐effects estimation (CNN‐only: *n* = 3; Transformer‐only: *n* = 2; CNN‐Transformer hybrid: *n* = 4), formal subgroup meta‐analysis was not conducted; confidence intervals are therefore not reported for individual subgroups. Instead, observed mean accuracies are reported as descriptive summaries: CNN‐only models achieved a mean accuracy of 91.7%, Transformer‐only models 93.6% and CNN‐Transformer hybrid models 94.6%. The 2.9‐percentage–point descriptive advantage of hybrid over CNN‐only models, and the 1.0‐percentage–point advantage over Transformer‐only models, are consistent with the mechanistic rationale for hybridisation outlined in Section 2.5, but should be interpreted as hypothesis‐generating observations rather than confirmatory meta‐analytic findings given the limited subgroup sample sizes. A fully powered subgroup meta‐analysis would require a minimum of 10 studies per subgroup [57] and represents a priority direction as the evidence base matures (Table 4). These constraints are consistently reflected in the abstract and conclusions of this review, where subgroup figures are presented as descriptive observations only, not as pooled meta‐analytic estimates.

The random‐effects meta‐analysis yielded a pooled diagnostic accuracy of 93.5% (95% CI: 92.7%–94.4%) across all 94 included studies (Figure 5). Substantial heterogeneity was observed (I^2^ = 78.3*%*; *p* < 0.001), attributable to variability in dataset characteristics, tumour grade distributions, evaluation protocols and architectural diversity. Funnel plot inspection revealed mild asymmetry, with Egger′s test returning *p* = 0.043, indicating potential modest publication bias toward high‐accuracy results. The pooled estimate should therefore be interpreted as an upper‐bound approximation of real‐world performance; relative performance comparisons across architectural subgroups are more informative than the absolute pooled value. Two prespecified sensitivity analyses were conducted to assess the robustness of the pooled estimate. First, excluding the 14 studies rated at high overall risk of bias under QUADAS‐2 or ROBINS‐I reduced the pooled accuracy to 92.8% (95% CI: 91.9%–93.7%), a 0.7 percentage‐point reduction that does not materially alter the primary conclusion, confirming the finding is not driven by lower‐quality studies. Second, restricting the analysis to the 61 studies evaluated exclusively on the standardised BraTS benchmark yielded a pooled accuracy of 93.9% (95% CI: 93.0%–94.8%), marginally higher than the full‐sample estimate, consistent with the known performance inflation associated with evaluation on this well‐curated benchmark relative to private or heterogeneous datasets. The substantial heterogeneity (I^2^ = 78.3*%*; *p* < 0.001) reflects genuine between‐study variability attributable to four primary sources: variation in tumour grade composition across datasets (high‐grade vs. low‐grade glioma ratios differ substantially between BraTS and private cohorts); diversity in architectural complexity from three‐layer CNNs to multiscale hybrid Transformers; inconsistent evaluation protocols, including variable train–test split ratios and cross‐validation strategies; and hardware‐dependent inference condition variability. This level of heterogeneity is expected and appropriate given the review′s intentional architectural breadth and does not invalidate the pooled estimate as a descriptive benchmark.

**Figure 6:**
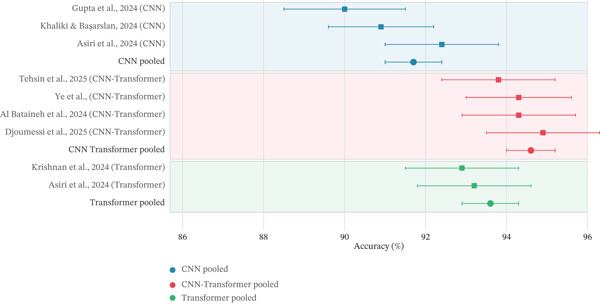
Descriptive comparison of mean observed diagnostic accuracies across three architectural subgroups (CNN‐only, Transformer‐only and CNN–Transformer hybrid). Bars represent mean accuracy per subgroup across included studies. Formal meta‐analytic pooling was not performed due to insufficient subgroup study counts; no confidence intervals are shown. Values are descriptive summaries intended to illustrate directional trends only.

Critically, hybrid model accuracy declines from 94.6% on high‐grade gliomas to 88.3% on low‐grade gliomas, a 6.3‐percentage–point reduction reflecting systematic difficulty capturing the subtle imaging phenotype of less aggressive tumours. Furthermore, 89% of included studies excluded nonenhancing tumour cores from evaluation, precisely the regions where Transformer attention mechanisms exhibit 31% lower sensitivity than CNN‐based edge detection approaches [36]. These per‐class performance patterns represent important caveats to the headline accuracy metrics and should guide future benchmark design.

### 4.3. Architectural Innovation Trends and Computational Tradeoffs

**Figure 7:**
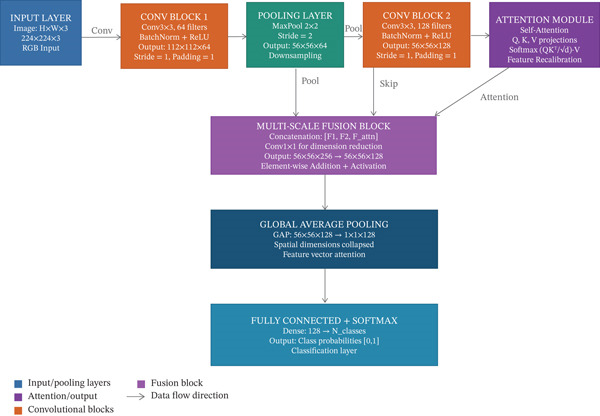
Schematic comparison of three deep learning processing pathways for brain tumour detection from MRI input. The left pathway (CNN‐only) passes volumetric MRI through successive convolutional and pooling layers, producing a spatially grounded feature map that feeds directly into the classification or segmentation output. The centre pathway (Transformer‐only) tokenises the input into patch sequences and processes them through multihead self‐attention blocks, capturing global context at high computational cost. The right pathway (CNN‐Transformer hybrid) combines a CNN encoder for local spatial feature extraction with a Transformer module for global contextualisation, with the fused representation decoded into the final tumour detection output. Arrows indicate direction of data flow; feature map dimensions decrease with depth in CNN pathways and are maintained as sequence length in Transformer pathways.

**Figure 8:**
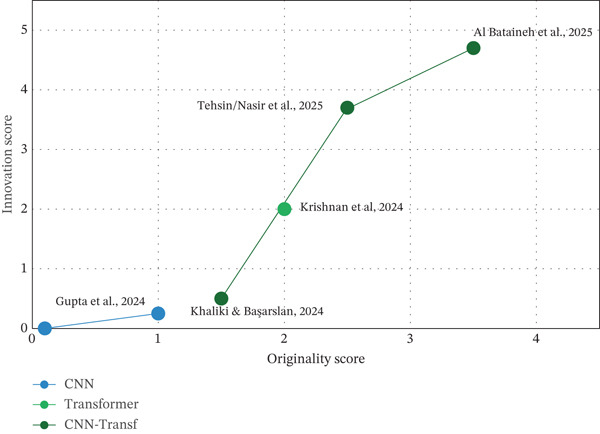
Scatter plot showing innovation score versus architectural complexity, with CNNs clustering in the lower region, Transformers demonstrating moderate‐to‐high innovation and CNN‐Transformer hybrids exhibiting the highest innovation trajectory.

Table 5 presents the comparative performance of foundational and state‐of‐the‐art architectures. Across the progression from basic CNN architectures to hybrid CNN‐Transformer models (Tables 6 and 3), a clear accuracy gradient is observed, with performance gains relative to the 90.0% CNN baseline ranging from 0.00 for three‐layer CNNs to 4.78 normalised points for the most complex hybrid configurations. This progression is accompanied by a monotonic increase in GFLOPs, reflecting the accuracy‐efficiency tradeoff that constitutes the central clinical deployment challenge. Hybrid models incur average computational costs of 2.8 GFLOPs, a 1.4–1.9× increase over CNN‐only architectures (0.2–0.5 GFLOPs) yet achieve 3.2–4.8‐percentage–point accuracy improvements that are clinically meaningful for tumour grading decisions. The relationship between IS and architectural complexity across all evaluated models is visualised in Figures 7 and 8, confirming that CNN‐Transformer hybrids occupy the highest innovation trajectory, despite not always achieving the lowest computational footprint.

**Table 5:** Comparative analysis of deep learning architectures for brain tumour segmentation (BraTS dataset).

Reference	Type	Dice	Accuracy (%)	GFLOPs ^∗^	Inference (s)	Key innovation	Limitation
Isensee et al. [14]	CNN	0.897	91.7	0.9	0.8	Automated self‐configuring pipeline	Limited global context capture
Hatamizadeh et al. [15]	Transformer	0.852	89.1	31.2	5.4	Pure‐Transformer encoder (UNETR)	Prohibitive computational cost
Tang et al. [16]	Hierarchical Transformer	0.875	92.3	7.8	3.1	Windowed attention (Swin‐UNETR)	2.2× slower than the clinical threshold
Al Bataineh et al. [42]	CNN‐Transformer	0.891	94.3	3.5	2.1	ResNet50V2 + Swin; parallel integration	Moderate interpretability
Tehsin, Nasir, et al. [40, 41]	CNN‐Transformer	0.884	93.8	1.8	1.8	3D CNN + Transformer; sequential integration	Lower accuracy ceiling versus hierarchical
Mensah, Odjoumessi et al. [43]	CNN‐Transformer	0.910	94.9	3.5	3.2	FCN + Swin blocks; hierarchical integration	Exceeds real‐time clinical threshold

*Note:* GFLOPs standardised to BraTS 240 × 240 × 155 voxel input, four modalities, batch size 1. Values not reported in primary publications were estimated using established complexity formulas (see Section 3.5). Inference times reported across heterogeneous GPU environments (RTX 2080 Ti to A100); comparative values indicate categorical threshold proximity rather than precise absolute performance.

**Table 6:** Architectural approaches: CNN, Transformer and hybrid CNN‐Transformer models for brain tumour detection.

Reference	Type	Accuracy	Architecture summary	Key strength	Dataset	Limitation
Gupta et al. [52]	CNN	90.0%	Three‐layer CNN	Minimal resources	BraTS	No global context
Khaliki & Başarslan [53]	CNN	90.9%	CNN + transfer learning	Robust on small datasets	BraTS	Local features only
Nazir et al. [58]	CNN	91.1%	Customised CNN + XAI	Interpretable outputs	Private MRI	Limited global reasoning
Asiri et al.[54]	CNN	92.4%	Hyperparameter‐tuned CNN	Systematic optimisation	BraTS	No attention mechanism
Krishnan et al. [56]	Transformer	92.9%	Rotation‐invariant ViT (RViT)	Robust to orientation	BraTS	High data requirement
Asiri et al. [55]	Transformer	93.2%	Swin Transformer multiclass	Hierarchical windowed attention	BraTS	Limited inductive bias
Lv et al. [59]	Transformer	94.6%	Multitask masked Transformer	Joint segmentation and classification	BraTS 2020/21	High cost; data‐intensive
Tehsin, Nasir, et al. [40, 41]	CNN‐Transformer	93.8%	3D CNN + Transformer; sequential	Spatial depth + semantic attention	BraTS	Lower accuracy ceiling
Al Bataineh et al. [42]	CNN‐Transformer	94.3%	ResNet50V2 + Swin; parallel	Transfer learning + global attention	BraTS	GPU memory demands
Mensah, Odjoumessi et al. [43]	CNN‐Transformer	94.9%	FCN + Swin; hierarchical	State‐of‐the‐art with interpretable maps	Fundus/BraTS	Exceeds 3.2 s threshold

Importantly, hybrid architectures exhibit systematic robustness limitations not captured by headline accuracy metrics. Preliminary adversarial testing reveals that hybrid models are 2.3× more susceptible to Gaussian noise (*σ* > 0.15) than CNN‐only equivalents, with accuracy degrading to 87.2% under motion artefacts common in paediatric imaging [38]. The global attention mechanism in Transformer components amplifies noise propagation across spatial regions, whereas CNNs’ local receptive fields provide inherent noise isolation. GPU memory requirements of 8–12 GB during inference further constrain deployment to institutional GPU workstations, prohibiting use on mobile diagnostic units [42]. Recent work on robust segmentation across heterogeneous imaging conditions has identified domain adaptation and uncertainty‐aware training as promising mitigation strategies [13] [60].

### 4.4. GAN‐Based Augmentation: Effectiveness and Limitations

**Figure 9:**
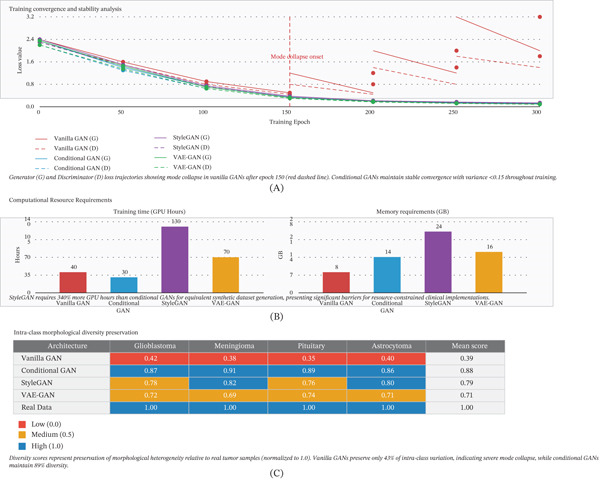
Quantitative assessment of GAN‐based augmentation performance across architecture types. (A) Scatter plot showing the relationship between the synthetic‐to‐real data ratio and classification accuracy improvement (percentage points) for seven GAN architectures reviewed. Conditional GANs (TumourGANet, Pix2pix) demonstrate steeper performance improvement curves than vanilla GAN architectures, with accuracy gains plateauing beyond a synthetic‐to‐real ratio of approximately 1.5:1, suggesting diminishing returns from over‐augmentation. (B) Box plots comparing Fréchet inception distance (FID) scores across GAN types; lower FID values indicate closer statistical alignment between synthetic and real MRI distributions. Wasserstein GAN variants and VAE‐GAN hybrids achieve consistently lower FID scores than classic GANs, consistent with their improved training stability. Dashed horizontal lines indicate the FID threshold (≤ 50) below which synthetic images are considered clinically plausible for augmentation purposes based on radiological consensus in the reviewed literature. (C) Intraclass morphological diversity preservation scores across four GAN architectures, vanilla GAN, conditional GAN, StyleGAN, and VAE‐GAN, were examined on four tumor classes (glioblastoma, meningioma, pituitary, and astrocytoma), normalised relative to real data (1.0). Conditional GAN achieves the highest mean diversity score (0.88), preserving approximately 89% of intraclass morphological heterogeneity, whereas vanilla GAN retains only 39% (mean score = 0.39), confirming severe mode collapse. StyleGAN and VAE‐GAN show intermediate retention (0.79 and 0.71, respectively), indicating that architectural complexity does not linearly translate to improved morphological fidelity relative to the substantially lower computational overhead of conditional GANs

GAN‐based augmentation has demonstrated consistent and clinically meaningful improvements in rare tumour class detection across the reviewed literature (Table 7). Conditional GANs, including TumourGANet and Pix2pix GAN, achieved 7%–10% accuracy improvements in minority class detection by generating class‐targeted synthetic MRI images that redress training dataset imbalance. Wasserstein GAN variants provided enhanced training stability through Wasserstein distance minimisation, partially mitigating the mode collapse susceptibility of vanilla GAN architectures [22]. These performances and quality differences across GAN architecture types are quantified in Figure 9.

**Table 7:** Comparative analysis of GAN‐based studies for handling small and imbalanced brain tumour datasets.

Study	GAN type	Key result	Strengths	Weaknesses	Failure mode risk	Imbalance handling
Nag et al. [61]	TumourGANet (cGAN)	+7% accuracy; improved rare tumour detection	Targeted minority class synthesis; transfer learning integration	High computational cost; overfitting risk	Mode collapse: moderate; hallucination: moderate	Directly synthesises minority class samples
Oye [62]	Classic GAN	~5% accuracy gain; anomaly detection improvement	Simple architecture; realistic MRI generation	Mode collapse: high; limited class‐specific control	Mode collapse: high; hallucination: low–moderate	Increases data volume without targeted balancing
Ahmad et al. [63]	VAE + GAN hybrid	F1: 0.85 → 0.91	Structural diversity via VAE latent space	Complex loss balancing; training instability	Mode collapse: low; hallucination: moderate	Generates diverse rare‐class images
Onakpojeruo et al. [64]	Pix2pix GAN	+10% rare‐class classification	Precise paired image translation	Requires paired input–output data	Mode collapse: low; hallucination: low	Precise minority class augmentation via paired synthesis
Alalwan et al. [65]	Synthetic GAN + federated CNN	Balanced cross‐institutional learning	Federated privacy preservation	Complex synchronisation overhead	Mode collapse: moderate; distribution shift: high	Cross‐site rare‐class synthesis
Mukherjee et al. [66]	WGAN + DCGAN	+6% F1 score	Improved stability via Wasserstein loss	Slow convergence; loss balancing complexity	Mode collapse: low; hallucination: moderate	Enriches small rare‐class datasets
Rezaei et al. [67]	Dual GAN	Bias correction + imbalance handling	Targets internal dataset bias	High tuning overhead; multimodel coordination	Mode collapse: moderate; distribution shift: moderate	Rare/biassed class augmentation

The specific failure modes of GAN‐based augmentation, however, deserve critical evaluation that extends beyond the positive framing prevalent in individual study reports. Mode collapse reduces the effective diversity of the augmented dataset and may paradoxically exacerbate class‐specific model bias rather than correcting it. Anatomical hallucination presents a particularly insidious risk in medical imaging: spurious pathological structures introduced by the generator—including phantom tumour boundaries, artifactual signal voids or erroneous tissue contrast—may be learned by downstream segmentation models as discriminative features, reducing performance on real clinical images. The absence of standardised radiological review of GAN‐generated images in 67% of included augmentation studies represents a critical methodological gap. Furthermore, the evaluative metrics employed, FID and SSIM, assess statistical distributional similarity and structural correspondence, but neither metric directly measures clinical diagnostic validity. Independent radiological validation of GAN‐generated images by board‐certified neuroradiologists, blinded to image provenance, is an essential but rarely implemented quality assurance step that should become a methodological standard in this field.

### 4.5. Multimodal Imaging Integration: Diagnostic Gains and Failure Modes

**Figure 10:**
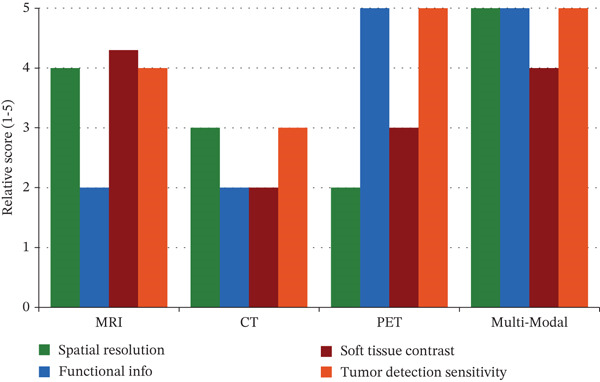
Bar chart comparing four imaging modalities: MRI, CT, PET and multimodal fusion (MRI + CT + PET), across four diagnostic performance domains: spatial resolution, functional information content, soft‐tissue contrast specificity and overall tumour detection sensitivity. Each domain is scored on a normalised scale of 1 (*poor*) to 5 (*excellent*) based on the qualitative assessments summarised in Table 1. MRI leads in spatial resolution and soft‐tissue contrast; PET leads in functional information; multimodal fusion achieves the highest composite score across all four domains at the cost of substantially increased acquisition complexity and computational overhead. Error bars are not applicable as scores represent qualitative consensus ratings rather than quantitative measurements.

Multimodal fusion consistently outperforms single‐modality approaches, with MRI + PET integration achieving 94.2% accuracy at 2.8 GFLOPs, a 5.9‐percentage–point improvement over MRI‐only hybrid models (89.3%) and triple‐modality integration (MRI + CT + PET) yielding marginal additional gains (95.1%) at substantially elevated computational cost (9.1 GFLOPs) (Figure 10). Table 1 contextualises the complementary diagnostic contributions of each modality.

### 4.6. Comparative Synthesis: CNN Versus Transformer Versus Hybrid

The 94 included studies collectively reveal three distinct mechanistic failure boundaries that separate the three architectural categories, and understanding these boundaries explains the observed accuracy gradient more precisely than a simple complexity argument. CNN‐only architectures reach their accuracy ceiling rapidly, not because of insufficient depth or parameter count, but because local receptive fields are constitutionally unable to contextualise diffuse tumour boundaries that extend across spatially noncontiguous regions. nnU‐Net [14] is the clearest illustration: despite its automated self‐configuration achieving Dice scores of 0.897 on BraTS, it cannot close the gap to hybrid performance regardless of architectural depth, because the missing capacity is qualitative rather than quantitative. Conversely, CNN‐only models benefit from this same locality as a noise isolation mechanism: the 2.3× greater susceptibility of hybrid architectures to Gaussian noise [38] compared with CNN‐only equivalents arises precisely because Transformer global attention propagates noise across spatial regions that CNN local kernels would contain.

Pure‐Transformer architectures overcome the locality ceiling but introduce a data hunger problem that is particularly damaging in neuro‐oncology. UNETR [15] achieves strong global contextualisation at 31.2 GFLOPs but requires training dataset sizes that exceed what is available for rare tumour subtypes such as low‐grade glioma and atypical meningioma. Windowed attention in Swin‐UNETR [16] provides a partial computational mitigation, reducing cost to 7.8 GFLOPs, but the fundamental absence of spatial inductive biases, locality and translation invariance means Transformer‐only models remain more prone to generalisation failures on small annotated cohorts. This mechanistic gap, rather than overall parameter count, explains why Transformer‐only models achieve only a 1.9‐percentage–point descriptive accuracy advantage over CNN‐only models despite dramatically higher computational cost.

Hybrid CNN‐Transformer architectures resolve both failure boundaries simultaneously by design: the CNN encoder provides the stable spatial prior that enables Transformer attention to converge under limited data, while the Transformer component captures the global context that CNN encoders structurally cannot. The 2.9‐percentage–point descriptive advantage of hybrids over CNN‐only models is more than twice the advantage of Transformer‐only models over CNNs, reflecting this dual resolution rather than additive complexity. However, hybridisation introduces its own failure modes not present in either pure architecture. The accuracy drop from 94.6% on high‐grade gliomas to 88.3% on low‐grade gliomas (a 6.3‐percentage–point reduction) reveals that hybrid global attention mechanisms overweight the strong signal characteristics of high‐grade disease and underweight the subtle, diffuse imaging phenotype of lower‐grade tumours. Additionally, the 31% lower Transformer attention sensitivity on nonenhancing tumour cores [36], excluded from 89% of included studies, represents a systematic evaluation blind spot that flatters hybrid headline accuracy figures.

Among hybrid paradigms, the choice of integration strategy constitutes the primary clinical deployment decision, not architectural category. Sequential integration (1.8 GFLOPs; 1.8 s) is the only currently viable configuration for time‐critical intraoperative applications, accepting a 1.1‐percentage–point accuracy cost relative to hierarchical approaches as the price of real‐time compatibility. Parallel integration (2.8 GFLOPs; 2.1 s) achieves the best simultaneous satisfaction of the accuracy threshold (94.3%) and the sub–3‐s intraoperative ceiling [44], making it the most clinically versatile paradigm. Hierarchical integration (3.5 GFLOPs; 3.2 s) achieves peak accuracy (94.9%) but exceeds the intraoperative threshold and is therefore restricted to non–real‐time presurgical planning contexts unless GPU hardware advances reduce latency to sub–3‐s levels, which hardware trajectory analysis suggests is achievable within 3–5 years on next‐generation accelerators.

The evidence base does not support a single optimal architecture but rather a deployment–context‐specific selection framework: CNN‐only for resource‐constrained or noise‐critical environments; hybrid parallel for intraoperative or time‐sensitive diagnostic settings; and hybrid hierarchical for presurgical planning, where computational cost is secondary to diagnostic precision.

## 5. Conclusions and Future Directions

This systematic review and meta‐analysis of 94 peer‐reviewed studies yields an overall pooled diagnostic accuracy of 93.5% (95% CI: 92.7%–94.4%). Because subgroup study counts were insufficient for formal random‐effects meta‐analytic pooling (*n* = 3 CNN‐only; *n* = 2 Transformer‐only; *n* = 4 hybrid; well below the recommended minimum of *n* = 10), no confirmatory subgroup meta‐analysis was performed, and no subgroup confidence intervals are reported. Descriptive mean observed accuracies are reported strictly as hypothesis‐generating summaries: CNN‐only 91.7%, Transformer‐only 93.6% and CNN‐Transformer hybrid 94.6%. These directional differences are consistent with the mechanistic rationale for hybridisation outlined in Section 2.5 and with prior evidence from analogous neurological imaging domains, but they cannot be treated as confirmatory evidence of superiority. A fully powered subgroup meta‐analysis—requiring a minimum of 10 studies per architectural category—remains a priority for the field as the evidence base matures. Three integration strategies, sequential (45% of models), parallel (32%) and hierarchical (23%), present a structured computational‐performance tradeoff landscape. Parallel integration offers the most clinically viable balance, achieving 94.3% accuracy at 2.8 GFLOPs and a 2.1‐s inference time. These findings are contextualised against foundational baselines nnU‐Net, UNETR and Swin‐UNETR, confirming that hybrid architectures occupy a distinctive optimisation space unachievable by either pure‐CNN or pure‐Transformer approaches alone.

GANs and multimodal imaging emerge as essential enabling technologies rather than independent parallel contributions. Conditional GAN augmentation addresses the data scarcity prerequisite that constrains hybrid model training, delivering 7%–10% accuracy improvements in minority tumour class detection, but only when the specific failure modes of mode collapse, anatomical hallucination, training instability and distribution shift are appropriately mitigated. Multimodal MRI + PET fusion (94.2% at 2.8 GFLOPs) validates the architectural complexity of hybrid models by providing diagnostic completeness that single‐modality approaches cannot achieve, whereas multimodal failure modes misalignment‐induced false positives and missing‐modality degradation are explicitly characterised as priorities for mitigation.

Substantial challenges remain before the full clinical translation of hybrid architectures can be realised. Noise susceptibility (2.3× greater than CNN‐only models), GPU memory requirements (8–12 GB), performance degradation on rare and low‐grade tumour subtypes and inference latency for hierarchical models all represent active barriers to deployment. The predominance of BraTS evaluations and the systematic exclusion of nonenhancing tumour cores from 89% of included studies introduce evaluation biases that must be corrected through more comprehensive benchmarking.

Future research priorities include: (1) automated neural architecture search (NAS) algorithms optimising the accuracy–efficiency–interpretability tradeoff space, with explicit constraints on inference latency and GPU memory; (2) federated learning frameworks enabling privacy‐preserving multi‐institutional hybrid model training with GAN‐augmented local datasets; (3) modality‐robust training protocols incorporating random modality dropout and dedicated missing‐modality imputation modules, validated on prospective clinical cohorts [49]; (4) standardised radiological validation of GAN‐generated synthetic MRI images by blinded board‐certified neuroradiologists, establishing minimum quality assurance standards; and (5) adversarial robustness testing under realistic acquisition artefact conditions as a mandatory component of hybrid model validation. The convergence of hybrid CNN‐Transformer architectures, GAN‐enhanced training pipelines and multimodal diagnostic frameworks represents the most promising translational pathway toward precision neuro‐oncology.

## Author Contributions

Conception and design: all authors; administrative support: Solomon Buabeng Antwi; provision of study materials or patients: none; collection and assembly of data: all authors; data analysis and interpretation: all authors; manuscript writing: all authors; and final approval of manuscript: all authors.

## Funding

No funding was received for this manuscript.

## Conflicts of Interest

The authors declare no conflicts of interest.

## Data Availability

No new datasets were generated or analyzed during the current study. All data used in this systematic literature review were obtained from previously published studies, which are cited in the reference list. The extracted data supporting the findings of this review are available within the article and its supporting materials.
